# Trauma, coherence, and vigilance across scales: a fractal regulatory framework for public mental health and preparedness

**DOI:** 10.3389/fpubh.2026.1794313

**Published:** 2026-03-20

**Authors:** Lawernce M. Nelson

**Affiliations:** Mary Elizabeth Conover Foundation, Inc., McLean, VA, United States

**Keywords:** complex adaptive systems, crisis governance, emergency preparedness, health system resilience, public mental health, regulatory coherence, systems coordination, trauma

## Abstract

Public health systems increasingly operate under conditions of recurrent, overlapping crises, challenging their technical capacity and their ability to sustain coordinated vigilance and recovery. While individual trauma is extensively examined, its relevance to population mental health and civic preparedness is less frequently integrated across biological and institutional domains. This Perspective proposes a biologically informed, systems-oriented framework that conceptualizes trauma as a disruption of regulated coherence within complex adaptive systems. Drawing on nervous system regulation and health system resilience, the framework advances the organizing concept that core regulatory processes—differentiation, integration, and phase-sensitive coordination—recur across levels of organization. This cross-scale recurrence is an analytic analogy, not a claim of structural equivalence. Three propositions structure the argument: (1) trauma is a loss of regulated coherence rather than exposure alone, with recovery involving integrated regulation; (2) civic domains are specialized regulatory subsystems whose coordination under stress shapes collective stability or fragmentation; and (3) shared regulatory principles can support repair-oriented coordination over time without reliance on sustained centralized control. An illustrative vignette clarifies the operational logic of phase-sensitive coordination in an emergency. By reframing vigilance as a regulatory capacity—sustaining sensitivity to threat while preserving integration and repair—this framework offers a unifying conceptual lens for examining how institutional coherence influences public mental health outcomes during prolonged crises. Although conceptual, it highlights measurable domains for future empirical study, including coordination transitions, information flow, and repair processes, suggesting implications for preparedness planning and institutional design.

## 1 Introduction

Public mental health refers to population-level conditions, determinants, and regulatory capacities that shape mental well-being across communities and institutions. It extends beyond the diagnosis and treatment of mental disorders to include prevention, promotion, resilience, and the structural features of social systems that influence psychological functioning across communities (1, 2). This distinction is critical. When trauma is conceptualized systemically, public mental health is best analyzed as a property of collective systems, not solely of individuals.

Public health systems are increasingly shaped by exposure to recurrent, overlapping crises, including infectious disease outbreaks, climate-related events, economic shocks, and large-scale social disruption. These conditions challenge not only surge capacity or technical response, but the ability of institutions to sustain vigilance, coordination, and repair over time. As the United States approaches its 250th anniversary since the Declaration of Independence, a milestone within settler history, it is important to acknowledge that Indigenous nations have sustained governance systems on this land for millennia, shaping insights into collective responsibility and trauma (3). A complementary focus could shift from founding ideals alone to the regulatory capacities that enable endurance: how societies detect threats, integrate information, coordinate action, and recover without fragmenting trust or function. Across these literatures, a shared regulatory framework linking biological regulation, trauma dynamics, and civic coordination under stress remains insufficiently articulated. The framework developed here, therefore, conceptualizes vigilance not as heightened control but as a capacity for phase-sensitive coordination that preserves integration while maintaining responsiveness to threat.

Resilience is widely invoked in public health to describe adaptive capacity under stress, yet the concept is often operationalized inconsistently, spanning individual psychology, community assets, institutional preparedness, and governance (4–6). Parallel literatures address trauma, emergency preparedness, and complex adaptive systems, but these domains are not consistently integrated through a shared regulatory framework (7). Trauma is often treated as an individual or clinical phenomenon, while civic and institutional responses to crisis are analyzed primarily in terms of authority, compliance, or resource allocation (7, 8). This separation may limit the field’s ability to explain how dysregulated responses propagate across scales, shaping collective outcomes beyond the acute phase of a crisis.

At the individual level, trauma is increasingly understood as a disruption of regulated coherence within the nervous system rather than as exposure alone (9–11). Adaptive functioning can be understood as depending on the integration of differentiated subsystems responsible for perception, threat detection, regulation, and recovery (12–14). When threats overwhelm regulatory capacity, coherence is reduced, possibly leading to fragmented or rigid responses that may persist after danger has passed. This model frames recovery as regulation and integration rather than control, and recovery as the restoration of safe, flexible coordination rather than suppression of threat signals.

Public health and preparedness research has independently advanced related insights (7, 8). Frameworks grounded in complex adaptive systems theory describe resilience as an emergent property of interacting subsystems shaped by information flow, feedback loops, and coordination under uncertainty (15–17). Empirical studies of emergency response suggest that sustained centralization may impede adaptation, while uncoordinated decentralization may amplify confusion and harm (4, 5, 7). Effective responses can involve phase-sensitive coordination: temporary centralization to stabilize signals and align priorities, followed by distributed adaptation as situational awareness improves (18, 19). These insights, however, are not always connected explicitly to regulatory principles derived from biological systems.

This Perspective introduces a conceptual framework that interprets trauma as a disruption of regulated coherence within complex adaptive systems. Within this framework, core principles of nervous system regulation—differentiation, integration, and phase-sensitive coordination—are examined as potential organizing dynamics that may recur across biological and civic domains (7, 8). From this perspective, public health crises can be viewed as regulatory stressors that test the coherence of systems across scales. Whether collective responses amplify harm or support recovery may depend on how effectively differentiated systems maintain integration, phase sensitivity, and intelligibility under sustained threat.

The framework is organized around three propositions: first, that trauma across scales reflects loss of coherence among differentiated systems; second, that civic domains function as specialized regulatory subsystems whose coherence under stress determines collective resilience; and third, that shared trauma-informed regulatory principles can enable repair-oriented coordination even without sustained centralized authority. By articulating this fractal regulatory framework, the paper offers a biologically grounded lens for public mental health and civic preparedness, reframing vigilance as a regulatory capacity that can be sustained without eroding trust or autonomy over the long arc of crisis exposure.

## 2 Conceptual framework: trauma as disrupted regulated coherence

This framework conceptualizes trauma as a disruption of regulated coherence within complex adaptive systems, beginning with the human nervous system, and proposes an extension fractally to civic and institutional domains (7, 8). Rather than treating trauma solely as an outcome of adverse exposure or as an individual clinical condition, the framework emphasizes regulation, integration, and coordination as candidate organizing principles across scales. From this perspective, crises can be understood as regulatory stressors that test whether differentiated systems can maintain coherent functioning under sustained threat.

Fractal governance, as used here, refers to the recurrence of similar coordination problems across nested levels of organization—signal detection, integration across specialized units, phase-sensitive shifts in authority, and repair—within complex adaptive systems (7, 8). The claim is not that civic institutions replicate biological structure, but that many public systems are polycentric: composed of semi-autonomous centers of decision-making whose interactions shape coherence under stress, enabling coordination without permanent centralization. In this sense, “fractal” functions as a systems lens for cross-scale coordination dynamics, not as a geometric claim.

At the biological level, adaptive nervous system function can be described as dependent on the coordinated integration of differentiated subsystems involved in perception, threat detection, autonomic regulation, and recovery (12–14). These subsystems differ in structure and function, yet are integrated through dynamic regulatory processes that enable flexible responses to changing conditions. Under manageable threat, temporary shifts in arousal and prioritization support protection and adaptation. When threat exceeds regulatory capacity, however, coherence is disrupted: integration breaks down, responses become fragmented or rigid, and recovery processes are delayed or impaired. Trauma, in this framework, reflects not only exposure to threat but also difficulty restoring regulated integration following threat exposure.

Importantly, this regulatory model does not require framing adaptation as centralized control, as complex adaptive systems theory emphasizes distributed coordination rather than singular command authority (7, 8). Instead, effective regulation emerges from coordinated interaction among differentiated components, a principle well characterized in large-scale brain network models (11, 12). Adaptive regulation depends on flexible integration across autonomic and cortical systems (14), and recovery following stress reflects restoration of coherent network functioning (10, 13).

Public health systems can be analyzed as exhibiting analogous properties (15–17). They are composed of specialized domains—such as surveillance, clinical care, logistics, communication, and governance—that must coordinate under conditions of uncertainty and time pressure. Like biological systems, these domains are differentiated for functional efficiency, yet interdependent in practice. Crises can introduce regulatory load by increasing information volume, compressing decision timelines, and elevating stakes for decision-makers (15, 17). When coordination mechanisms fail, systems may oscillate between excessive centralization, which constrains local adaptation, and fragmented decentralization, which erodes coherence and intelligibility.

Complex adaptive systems theory provides a formal language for describing these dynamics, including cross-scale interactions that shape resilience across nested systems (7, 8). Within this literature, resilience is understood as an emergent property arising from interactions among system components, shaped by information flow, feedback loops, and adaptive capacity (15–17). Crucially, resilience does not reside in any single unit or authority, but in the quality of coordination across the system. Emergency response research and preparedness syntheses suggest a recurring coordination tradeoff: sustained centralization may constrain local adaptation, while poorly coordinated decentralization may fragment situational awareness and implementation (8, 17). This is presented as a coordination hypothesis consistent with accounts of complex adaptive systems, not as a universal empirical law.

**Figure 1:**
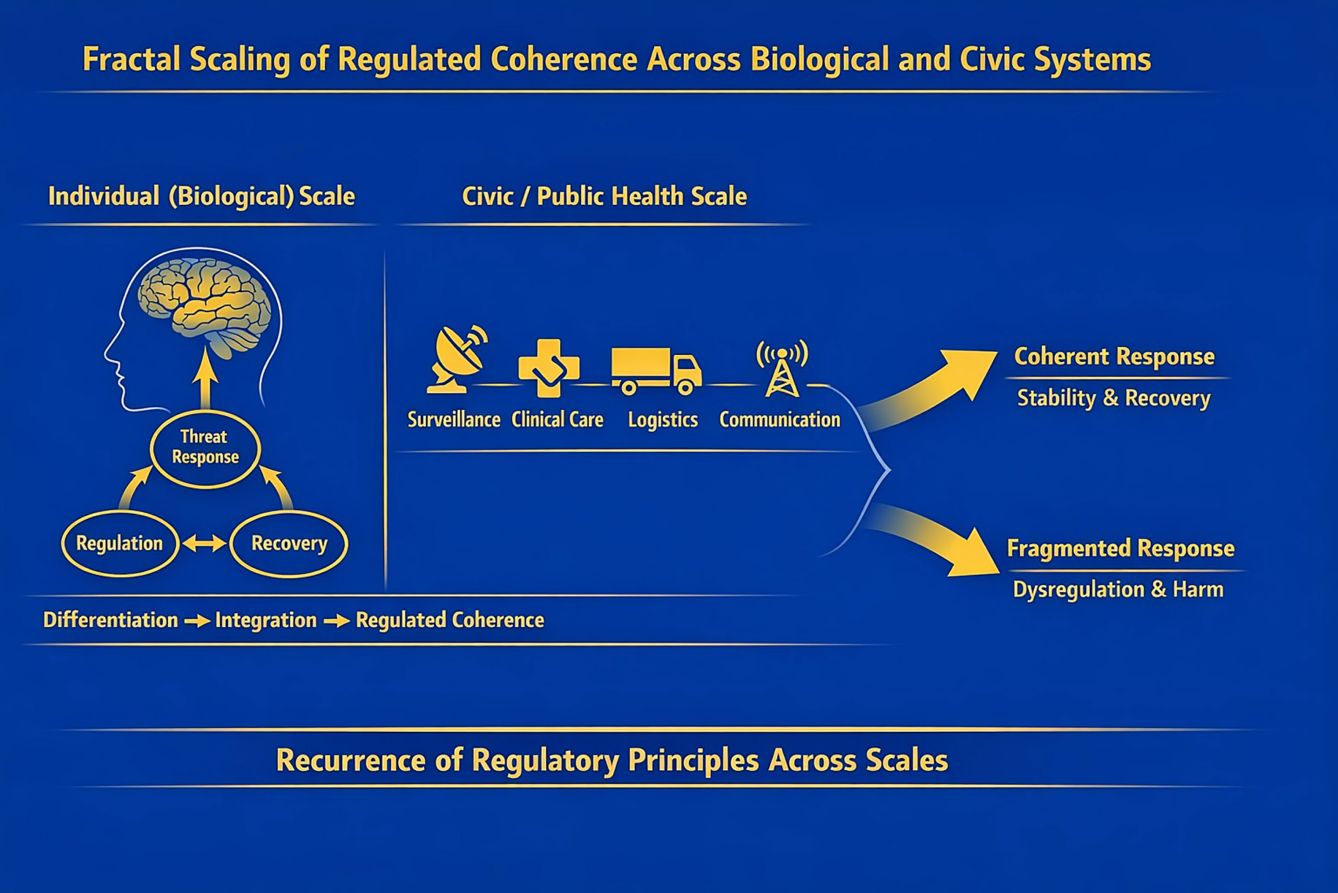
Fractal scaling of regulated coherence across biological and civic systems. The central conceptual framework is that principles of regulated coherence governing the human nervous system under threat scale fractally to civic and public health systems. At the individual level, adaptive functioning depends on the integration of differentiated regulatory subsystems (e.g., threat detection, regulation, recovery). At the civic level, specialized domains (such as surveillance, clinical care, logistics, communication, and governance) similarly function as differentiated regulatory subsystems whose coordinated integration determines system behavior under stress. Across scales, loss of regulated coherence leads to fragmentation or rigidity, while restoration of integration supports recovery and adaptive response. Fractality here refers to the recurrence of organizing regulatory principles across scales rather than structural equivalence.

These findings may parallel biological models in which temporary regulatory dominance gives way to reintegration once the threat subsides. Figure 1 summarizes the proposed fractal scaling of regulated coherence from nervous system dynamics to civic and public health domains.

The fractal concept advanced here is that similar regulatory coordination problems may recur across scales. Fractality, in this context, does not imply identical structures or simple replication, but the recurrence of organizing principles governing coherence under stress. Just as nervous system regulation can be understood as depending on differentiated subsystems coordinating through shared regulatory constraints, civic systems may depend on specialized domains coordinating through shared protocols, thresholds, and norms (6, 8, 11). Trauma at the civic level can thus be understood as a loss of regulated coherence among institutional domains.

This frames vigilance as a regulatory capacity rather than a state of heightened control (13, 14). Vigilance, in biological systems, involves sustained sensitivity to relevant signals while preserving the capacity for integration and recovery (11, 20). In public health, vigilance can entail maintaining clear coordination across domains over time, particularly as crises recur or overlap (5, 6, 15). From this perspective, the central challenge is not maximizing control or compliance, but sustaining conditions that support regulated integration under prolonged stress (8, 16).

The following propositions elaborate this framework by specifying how loss of coherence manifests across scales, how civic domains function as regulatory subsystems, and how shared regulatory principles can support repair-oriented coordination without sustained centralization (15–17). An illustrative vignette is included to demonstrate how these principles organize coordination within a public health emergency, clarifying the framework’s operational logic without claiming empirical validation.

### 2.1 Illustrative vignette: phase-sensitive coordination in a public health emergency

To clarify how the proposed framework organizes coordination under conditions of collective stress, the theme calls for a brief illustrative vignette. This vignette is intentionally composite and non-empirical, and is offered solely to demonstrate the operational logic of phase-sensitive regulation within a public health emergency rather than to claim effectiveness or generalizability (8). The 2014–2016 West African Ebola epidemic illustrates how regulatory coherence shifts across phases of crisis (6, 19).

A regional public health system confronts an unfamiliar infectious threat (21, 22). Often, early signals are fragmented: emergency departments report atypical cases, laboratories face testing uncertainty, and public communication is incomplete—patterns commonly observed in the response to emerging infectious diseases (21, 22). In the acute phase, temporary centralized coordination filters signals, aligns surveillance priorities, and stabilizes information flow. Distinct units—clinical care, epidemiology, logistics, and communications—retain specialized roles while operating under shared protocols.

As uncertainty decreases, coordination shifts (13, 20). Authority becomes more distributed, enabling domain-specific adaptation while maintaining coherence through common regulatory principles. Information flows horizontally, and repair-oriented functions— including workforce recovery, supply normalization, and trust maintenance—are reintegrated into planning. This vignette illustrates how phase-sensitive coordination can preserve system intelligibility and adaptive response without sustained centralized control.

**Figure 2:**
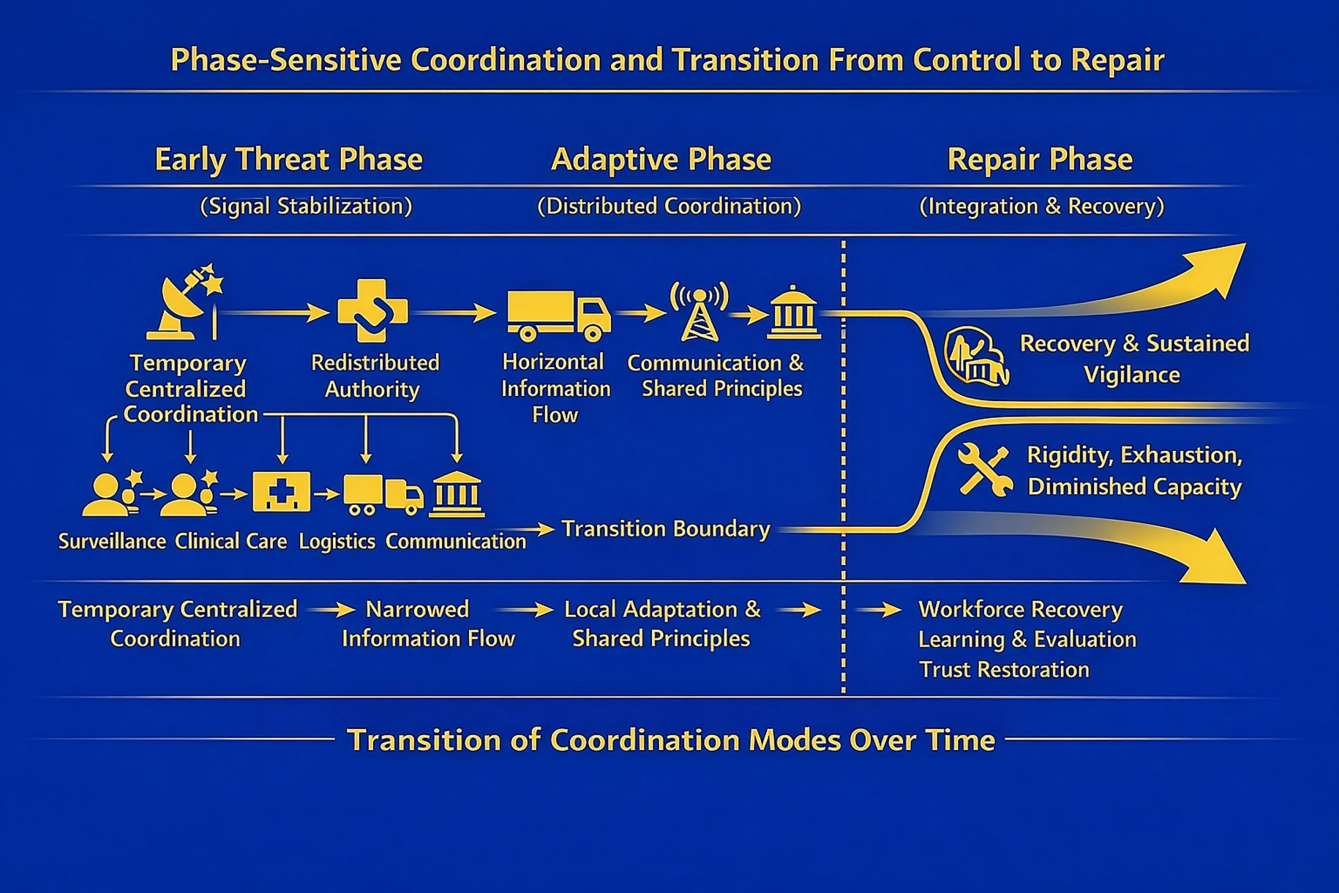
Phase-sensitive coordination and transition from control to repair. Phase-sensitive coordination during a public health emergency highlights how effective system regulation depends on transitions in coordination mode over time. In early threat phases, temporary centralized coordination supports signal stabilization, alignment, and containment. As uncertainty decreases, authority redistributes across domains, enabling horizontal information flow and local adaptation guided by shared regulatory principles. In later phases, repair-oriented functions—such as workforce recovery, learning, and trust restoration—are reintegrated to support sustained vigilance and recovery. Failure to transition between coordination phases risks rigidity, exhaustion, and loss of coherence, whereas successful transitions preserve intelligibility without reliance on sustained centralized control.

Figure 2 depicts the phase-sensitive transition from early signal stabilization to distributed adaptation and repair.

The following sections elaborate on the framework’s core propositions, specifying how loss of regulated coherence manifests across scales and how shared regulatory principles may support repair-oriented coordination within and across civic domains.

### 2.2 Proposition 1: trauma as loss of coherence across scales

The first proposition of the framework is that, across scales, trauma may be best understood as a loss of regulated coherence among differentiated systems, with recovery depending on the restoration of safe, integrated regulation rather than the assertion of control (8, 14). This proposition reframes trauma away from event-based or symptom-based definitions and toward a systems-level account of dysregulation that applies to both biological and civic contexts (3, 6).

At the level of the human nervous system, adaptive functioning depends on the coordination of specialized subsystems that are differentiated for efficiency yet integrated through dynamic regulatory processes (11, 14). Threat exposure temporarily alters this coordination, prioritizing rapid detection and protective responses (13). Under conditions of intense, prolonged, or unpredictable threat, regulatory capacity can be exceeded, leading to persistent disruptions in integration (20). Neurobiological research on post-traumatic stress has reported altered functional connectivity among regions involved in threat detection, memory, and regulation, suggesting that trauma may involve network-level dysregulation rather than isolated deficits (9, 10). From this perspective, symptoms can be interpreted as downstream expressions of reduced coherence rather than as primary targets of intervention (11).

Crucially, this model emphasizes that loss of coherence does not imply absence of function. Dysregulated systems may remain highly active, but activity may become fragmented, rigid, or poorly coordinated across regulatory subsystems (11, 14). Attempts to restore stability through increased suppression or top-down control may further reduce integration by constraining adaptive feedback and flexibility (13). Recovery, by contrast, involves re-establishing conditions that support safe, regulated integration across subsystems, allowing threat responses to resolve and regulatory balance to return (10, 20).

This logic extends fractally to civic and institutional systems (15–17). Public health crises introduce regulatory load by increasing informational uncertainty, compressing decision timelines, and elevating consequences of error (21, 22). Civic domains—such as surveillance, clinical care, logistics, and communication—are differentiated to manage these functions efficiently, yet must remain integrated to respond coherently. When coherence is lost, systems may exhibit patterns analogous to biological dysregulation: fragmented responses, rigid adherence to outdated protocols, or oscillation between over-centralization and uncontrolled decentralization. These patterns can persist beyond the acute phase of a crisis, shaping institutional behavior and public trust long after immediate threats subside.

Understanding trauma as a loss of coherence across scales clarifies why control-oriented responses can fail to produce durable recovery (13, 20). Just as biological systems recover through the restoration of regulated integration rather than prolonged defensive activation, civic systems recover through the re-establishment of intelligible coordination among differentiated domains. This framing shifts analytic attention from identifying single points of failure or authority toward examining how coordination, feedback, and regulatory flexibility are supported or constrained over time.

By grounding trauma in coherence rather than exposure or pathology alone, this proposition provides a unifying lens for linking individual and collective responses to crisis. It establishes the conceptual foundation for the subsequent propositions, which examine how civic domains function as regulatory subsystems and how shared regulatory principles can support repair-oriented coordination under sustained stress.

Why regulation, integration, and coordination? These principles are characterized in neuroscience and complex systems science and recur across levels of organization. Neural models describe adaptive regulation as the integration of differentiated networks responsible for threat detection, cognitive control, and autonomic regulation (11, 14), while complexity science similarly emphasizes coordination among interacting subsystems as the basis of adaptive system behavior (8).

### 2.3 Proposition 2: civic domains as specialized regulatory subsystems

The second proposition is that civic domains function as specialized regulatory subsystems whose coherence under stress determines whether collective responses to crisis amplify threat or provide stability (5, 6). This proposition extends the regulatory logic articulated at the biological level to public health and civic systems, emphasizing differentiation and integration as prerequisites for adaptive collective functioning (8, 11, 14).

Public health and civic systems are inherently differentiated (16, 17). Domains such as surveillance, clinical care, laboratory services, logistics, risk communication, and governance are organized to efficiently perform distinct regulatory functions (18, 19). This differentiation enables sensitivity to diverse signals, parallel processing of complex tasks, and specialization of expertise (8). However, differentiation alone is insufficient. As in biological systems, adaptive performance depends on the capacity of these subsystems to remain coherently integrated under conditions of uncertainty and time pressure (6, 15).

Crisis conditions place extraordinary regulatory demands on civic systems (15–17). Information volume increases rapidly, signals are often ambiguous or conflicting, and decision timelines are compressed. Under these conditions, coordination failures can manifest in predictable patterns. Excessive centralization may emerge as an attempt to restore control, narrowing information flow and constraining local adaptation. Conversely, uncoordinated decentralization may fragment responses, eroding shared situational awareness and public trust. Both patterns reflect loss of regulated coherence among subsystems rather than failure of any single domain.

Public health preparedness research increasingly conceptualizes resilience as an emergent property of interlinked subsystems rather than as a function of centralized authority or resource stockpiles alone (7, 8). Frameworks grounded in complex adaptive systems theory describe resilience as an emergent property of interacting subsystems linked through feedback loops and adaptive coordination (5, 7, 8). From this perspective, governance functions not as a singular command center but as a regulatory layer that shapes how subsystems interact, sets thresholds, and adjusts roles over time (18, 19).

Understanding civic domains as regulatory subsystems clarifies why sustained coherence is fragile under prolonged or overlapping crises (13, 20). When regulatory load remains elevated, coordination mechanisms themselves become stressed. Subsystems may compete for resources, operate under misaligned incentives, or revert to rigid routines that once served stability but now impede adaptation. These dynamics can persist beyond the acute phase of a crisis, contributing to institutional mistrust, workforce exhaustion, and diminished public confidence.

This proposition reframes the analytic task of public mental health and preparedness. Rather than asking which domain should dominate decision-making, it directs attention to how regulatory relationships among domains are structured and maintained. Coherence depends on shared protocols, intelligible information flows, and phase-appropriate shifts in authority that preserve both integration and differentiation. When these conditions are met, civic systems may be more likely to provide a safe harbor under stress—absorbing threat without amplifying it.

By conceptualizing civic domains as specialized regulatory subsystems, this proposition establishes the conditions under which coordination can support resilience rather than fragmentation. It sets the stage for the third proposition, which examines how shared trauma-informed regulatory principles can sustain repair-oriented coordination even in the absence of sustained centralized control.

### 2.4 Proposition 3: shared regulatory principles enable repair-oriented coordination without sustained centralization

The third proposition is that when trauma-informed regulatory principles are shared across civic domains, systems can maintain intelligibility and support repair-oriented coordination even in the absence of sustained centralized authority (5, 16). This proposition addresses how coherence can be preserved over time, particularly during prolonged or recurrent crises, without reliance on continuous top-down control (8, 15).

In both biological and civic systems, regulation under stress is inherently phase-sensitive (6, 13). Early phases of threat often require a temporary concentration of authority to stabilize signals, reduce noise, and establish shared priorities (15, 17). In nervous system regulation, this may involve transient dominance of threat-detection and protective responses (14, 20). In civic contexts, it may involve centralized incident command, emergency declarations, or unified communication strategies (18, 19). These mechanisms are adaptive when bounded in time and scope, supporting rapid alignment under uncertainty (6, 8).

However, prolonged centralization carries costs (8, 15). As threat conditions evolve, sustained concentration of authority can constrain local adaptation, suppress feedback, and erode trust (5, 17). Biological systems under prolonged defensive activation exhibit reduced flexibility and impaired recovery (13, 20); similarly, civic systems may experience workforce exhaustion, procedural rigidity, and declining public confidence under extended emergency governance (6, 16). Effective recovery, therefore, depends not on maintaining control but on transitioning coordination modes as conditions change (6, 8).

Shared regulatory principles provide the mechanism for this transition (5, 8). When domains share common understandings of thresholds, roles, feedback loops, and repair priorities, authority can be redistributed without loss of coherence (16, 17). Coordination becomes intelligible because actors are guided by shared constraints rather than constant directives (15, 18). Information continues to circulate horizontally, adaptations remain aligned with system-wide goals, and repair-oriented functions—such as workforce recovery, learning, and trust restoration—are reintegrated into operational planning (5, 6).

Research on multi-team systems and emergency coordination supports this logic (15, 23). Studies suggest that performance improves when coordination structures shift over time, moving from centralized control in early phases toward more distributed, horizontal coordination as situational awareness increases (15, 17). Importantly, successful transitions may depend less on formal hierarchy than on shared protocols, explicit role clarity, and common regulatory norms (16, 18). These findings parallel biological models in which recovery depends on re-establishing integrated regulation rather than prolonging defensive dominance (10, 13, 14).

From a public mental health perspective, this proposition highlights repair as an essential regulatory function rather than an afterthought. Repair involves restoring conditions that allow systems and individuals to recover flexibility, meaning, and trust following disruption. When shared regulatory principles foreground repair alongside protection, civic environments may be more likely to remain intelligible under stress, reducing the risk that crisis responses themselves become sources of ongoing trauma.

By emphasizing shared regulatory principles over sustained centralization, this proposition completes the framework’s account of how coherence can be maintained across scales (5, 8). Empirical research on emergency coordination further supports the importance of adaptive redistribution of authority and shared norms over rigid hierarchy (15–17). Together with the preceding propositions, this framework suggests that durable vigilance depends not on heightened control, but on the capacity of differentiated systems to coordinate, transition, and repair under prolonged stress (6, 13, 14). This framing sets the stage for considering the broader implications of coherence-based regulation for public mental health and preparedness.

## 3 Implications for public mental health and preparedness

Taken together, the preceding framework and propositions suggest a reframing of public mental health and preparedness that centers regulation, coherence, and repair rather than control, compliance, or crisis-specific optimization (5, 8). From this perspective, preparedness may not be solely a matter of technical capacity or resource availability, but may also be a function of how effectively systems maintain regulated integration under sustained or recurrent stress (6, 16). For example, the Mpox outbreak, declared a public health emergency of international concern, has important implications for mental health and global preparedness (21).

First, the framework implies that public mental health must be understood as an emergent property of coordinated system functioning rather than as an aggregate of individual psychological outcomes alone (21, 22). While individual-level interventions remain essential, population-level mental health during crises is shaped by the coherence of civic responses (6, 15). Fragmented communication, rigid protocols, or prolonged uncertainty can function as regulatory stressors in their own right and may amplify distress (2, 24). Conversely, intelligible coordination across domains can buffer stress, support meaning-making, and may reduce secondary traumatization (21, 25).

Second, preparedness efforts may benefit from explicit attention to phase-sensitive coordination (6, 8). Traditional preparedness models often emphasize command structures and predefined roles (18, 19), but may fail to adequately address how coordination should evolve over time (15). The framework proposed here highlights the importance of designing for transition: from early stabilization and signal filtering to later phases of distributed adaptation and repair (17, 23). Public health systems that lack mechanisms for transitioning between coordination modes may remain locked in defensive postures, potentially impairing recovery and organizational learning once acute threats subside (5, 16).

Third, the emphasis on shared regulatory principles points to the value of common protocols, thresholds, and norms that cut across domains (5, 16). Rather than relying on continuous central direction, systems might support coherence by aligning expectations about information sharing, decision thresholds, and repair priorities (17, 18). This alignment could enable authority to redistribute without loss of intelligibility, supporting adaptive responses at local levels while preserving system-wide coordination (15, 23). Importantly, such principles can be developed and practiced in advance, reducing regulatory load during crises (6, 8).

From a workforce perspective, the framework foregrounds repair as a core preparedness function (5, 6). Public health personnel and allied professionals are themselves subject to prolonged regulatory stress during crises, sometimes with limited opportunities for recovery (2, 13). When repair-oriented processes—such as rest, reflection, learning, and role recalibration—are deferred or treated as optional, systems risk cumulative exhaustion and loss of capacity (6, 16). Embedding repair into preparedness planning acknowledges that sustained vigilance depends on restoring regulatory balance rather than maintaining perpetual activation (14, 20).

Finally, this framework offers implications for institutional trust and public engagement. Public responses to crisis are shaped not only by policy decisions, but by how those decisions are communicated, coordinated, and revised over time (21, 22). Coherence-based approaches that preserve intelligibility and demonstrate adaptive learning may help maintain trust even amid uncertainty (5, 15). By contrast, oscillations between rigid control and uncoordinated relaxation can erode confidence and contribute to collective fatigue (13, 24, 25).

These implications do not prescribe specific policies or organizational forms. Rather, they provide a conceptual lens for evaluating preparedness strategies in terms of their regulatory effects across scales. By emphasizing coherence, phase sensitivity, and repair, the framework invites public mental health practitioners and preparedness planners to consider not only the actions taken during crises but also how those actions shape the system’s capacity to endure, adapt, and recover over time.

## 4 Discussion: vigilance, coherence, and the long arc of crisis

This Perspective advances a coherence-based framework for understanding trauma and recovery across scales, linking nervous system regulation to public mental health and civic preparedness. In doing so, it contributes to ongoing public health efforts to move beyond event-specific or siloed models of crisis response toward integrative approaches that may account for sustained regulatory load, coordination dynamics, and recovery over time.

A central contribution of the framework is its reframing of vigilance (13, 14). Vigilance is often implicitly equated with heightened monitoring, enforcement, or control (15). In contrast, the regulatory perspective proposed here conceptualizes vigilance as the capacity to sustain sensitivity to relevant signals while preserving integration, flexibility, and repair (11, 20). This distinction is consequential. Systems that equate vigilance with control may risk prolonged defensive activation, rigidity, and erosion of trust (13, 24). Systems that support regulatory vigilance may be better positioned to adapt, learn, and recover under recurrent or overlapping crises (5, 6, 16).

The framework seeks to clarify why public mental health outcomes cannot be fully understood through individual-level measures alone. While psychological distress and resilience are experienced by individuals, they are shaped by the regulatory environment in which individuals are embedded. Coherent coordination across civic domains can function as a protective factor, reducing secondary stressors and supporting meaning-making during crises. Conversely, fragmented or unintelligible responses may amplify distress, even when material resources are adequate. This insight reinforces calls within public health to attend to institutional and systemic determinants of mental health alongside individual interventions.

Several limitations warrant consideration. First, the framework is conceptual and does not claim empirical validation in its entirety. While each proposition is conceptually grounded, further work is needed to operationalize coherence, phase sensitivity, and repair in measurable terms at the civic and institutional levels. Second, the framework does not prescribe specific organizational forms or governance models. Its utility lies in offering a lens for analysis and design rather than a blueprint for implementation. Third, the fractal scaling claim should be understood as an analogy of organizing principles rather than a claim of structural equivalence across biological and civic systems.

Despite these limitations, the framework offers a generative platform for future research and practice. Empirical studies could examine how coherence-related variables—such as information flow, coordination transitions, and repair processes—relate to public mental health outcomes during and after crises. Comparative analyses across jurisdictions or sectors may further clarify how shared regulatory principles are established and sustained under different conditions.

As societies confront an era of recurring disruption, the challenge is not only to respond effectively to individual crises but to preserve the capacity for vigilance across time. By foregrounding coherence, phase-sensitive coordination, and repair, this framework suggests that endurance may depend less on escalating control than on sustaining regulated integration. In this sense, vigilance becomes not a posture of alarm, but a disciplined capacity for collective care under conditions of uncertainty.

## 5 Conclusion

This Perspective has offered a coherence-based framework for understanding trauma, recovery, and vigilance across biological and civic scales. By conceptualizing trauma as a disruption of regulated coherence within complex adaptive systems, the framework links established principles of nervous system regulation to public mental health and preparedness, offering a unifying lens for interpreting collective responses to crisis.

Across three propositions, the paper has posited that loss of coherence—rather than exposure alone—may provide a parsimonious account of dysregulation under sustained threat; that civic domains may function as specialized regulatory subsystems whose coordination determines whether crises amplify harm or support stability; and that shared trauma-informed regulatory principles may enable repair-oriented coordination without reliance on sustained centralized control. Together, these perspectives suggest that public mental health outcomes may be shaped not only by individual vulnerability or resilience, but by the regulatory quality of the environments in which individuals live and work.

Reframing vigilance as a regulatory capacity rather than a posture of control has practical implications for how preparedness is conceptualized and assessed. Endurance in the face of recurrent disruption may depend on maintaining integration, phase-sensitive coordination, and repair over time. Systems that preserve intelligibility and adaptive flexibility may be better positioned to support recovery without eroding trust or autonomy.

While the framework is conceptual, it offers a foundation for future empirical work and design-oriented inquiry. By foregrounding coherence as a central organizing principle, this Perspective invites public mental health researchers and practitioners to consider not only how crises are managed, but how systems are structured to sustain vigilance, care, and recovery across the long arc of uncertainty.

## Data Availability

The original contributions presented in the study are included in the article/supplementary material, further inquiries can be directed to the corresponding author.
